# A novel inactivated vaccine against *Lawsonia intracellularis* induces rapid induction of humoral immunity, reduction of bacterial shedding and provides robust gut barrier function

**DOI:** 10.1016/j.vaccine.2017.12.049

**Published:** 2018-03-07

**Authors:** F. Roerink, C.L. Morgan, S.M. Knetter, M.-H. Passat, A.L. Archibald, T. Ait-Ali, E.L. Strait

**Affiliations:** a Merck Animal Health, 2 Giralda Farms, Madison, NJ 07940, USA; b The Roslin Institute and Royal (Dick) School of Veterinary Studies, University of Edinburgh, Easter Bush Campus, Roslin, Midlothian EH25 9RG, UK

**Keywords:** Swine, Ileitis, *Lawsonia intracellularis*, Intestinal integrity, Vaccine, Efficacy

## Abstract

Porcine proliferative ileitis is a major economic burden for the swine industry, affecting growing pigs and young adult pigs. In this study, the protective efficacy of an inactivated, injectable whole-cell bacteria vaccine against *L. intracellularis* – Porcilis® Ileitis was evaluated under field conditions.

Eighty-five, three-week-old pigs on a commercial farrow-to-finish farm were vaccinated by the intramuscular route, either with a dose of injectable vaccine, or with saline. A subset of vaccinates and control pigs were necropsied at 21 days post-challenge. Incidence and severity of ileitis were evaluated by gross and microscopic observation of ileal tissues. Colonization of the gut after challenge was examined by *L. intracellularis*-specific immunohistochemistry, and qPCR of ileal scrapings. Integrity of the intestinal barrier was evaluated to quantify a range of intestinal markers including secreted mucin and intestinal alkaline phosphatase, and innate immune markers including Caspase-3 and Calprotectin. A second subset of pigs was monitored for fecal shedding of *L. intracellularis*, until resolution of shedding.

Our investigation indicated that Porcilis Ileitis provided robust protection against ileitis, reduced bacterial shedding 15-fold (*p* < .05) and preserved normal gut barrier function in the face of an experimental challenge with virulent *L. intracellularis*.

## 1 Introduction

Ileitis caused by *Lawsonia intracellularis* (*L. intracellularis*) continues to be a problem in swine production systems worldwide. *L. intracellularis* is a Gram-negative, intracellular bacterium that can infect a number of animal species, but it is of particular economic concern in the swine industry. In pigs, the bacteria cause porcine proliferative enteropathy (ileitis). Clinically affected animals exhibit diarrhea and reduced growth performance, resulting in increased time to market and greater variation in size between pigs. In young adults, the infection can lead to an acute hemorrhagic form of the disease, characterized by dark, tarry diarrhea and which may result in death. *L. intracellularis* also infects pigs sub-clinically, without clear clinical signs but still resulting in reduced growth performance. Its worldwide distribution and high prevalence have been recognized since the initial characterization of this pathogen in the early 1990s and *L. intracellularis* is reported to affect 57–100% of herds, globally [1], [2], [3].

As an obligate intracellular pathogen, interaction between *L. intracellularis* and host cells is crucial in establishing infection. The bacterium infects the gastrointestinal tract, with a specific tropism for the terminal ileum. The hallmark lesion of *L. intracellularis* infection is the proliferation of intestinal crypt lining cells (enterocytes) which results in hyperplasia of the mucosal wall. The peak of bacterial burden is associated not only with crypt epithelial cell proliferation but also with down-regulation of specific host mechanisms involved in cell transport and maintenance of mucosal integrity, and with inflammation [4], [5], [6]. It is likely that the poor performance and growth of affected animals are a direct consequence of these cell differentiation alterations [7].

There are some tools available for controlling *L. intracellularis* infections and limiting the associated economic losses. Infection by the bacterium can be treated with various antibiotics, notably those from the macrolide, pleuromutilin, and quinoxaline groups [7]. For prophylaxis, a modified live-attenuated vaccine has been commercially available since 2001 [8]. Due to the live nature of the oral vaccine, concurrent use with antibiotics effective against *L. intracellularis* is not possible. The use of the oral vaccine requires strict management practices to avoid the simultaneous use of antibiotic treatments. However, prophylactic use of an inactivated vaccine would not be limited in this way.

In this study, the effectiveness of a novel inactivated injectable vaccine, Porcilis Ileitis, as an aid in the control of ileitis caused by *L. intracellularis* was examined. This vaccine was administered to three-week-old pigs under typical field conditions, without restricting the use of antibiotics. Our investigation indicates that Porcilis Ileitis vaccine can provide robust protection against ileitis, help reduce bacterial shedding 15-fold (*p < .01*), and help maintain gut barrier function integrity.

## 2 Materials and methods

### 2.1 Ethical statement

The animal trial was conducted by Swine Services Unlimited (Rice, MN, USA, SSUI) as a randomized, blinded study, approved by the SSUI Institutional Animal Care and Use Committee.

### 2.2 Vaccine

The vaccine used in this study contained inactivated *L. intracellularis* bacteria in XSOLVE adjuvant (Porcilis Ileitis, serial 02381108, Merck Animal Health, Madison, NJ USA). The vaccine is an oil-in-water emulsion. The adjuvant is based on both mineral oil and alpha tocopherol (Vitamin E).

### 2.3 Study design

Eighty-five mixed-breed and mixed-sex pigs were enrolled on a commercial, farrow-to-finish farm, which farrowed 30 litters per week, weaning 300 pigs per week. The herd was health-stable prior to and during the study, with historic pre-weaning mortality rates between 8 and 10% during the previous 2 years, and nursery mortality of approximately 3%. The herd did not show clinical signs of *L. intracellularis* infection, and all study pigs were negative for anti-*L. intracellularis* antibodies as measured by a commercial inhibition ELISA (bioScreen Ileitis Antibody ELISA, Svanova, Sweden).

**Fig. 1:**
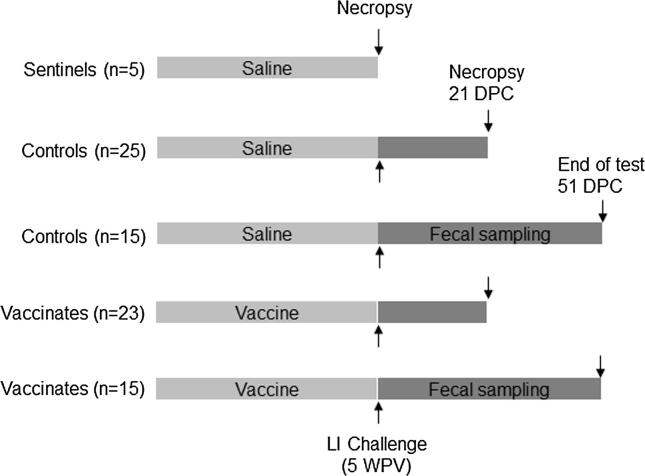
Study design. Read this figure in conjunction with supplementary Table 1 and the materials and methods section.

The study design is summarized in Fig. 1. Pigs were allocated to the treatments using a random number generator, so that both vaccinates and placebo injected controls were represented within all litters. At 22–25 days of age (23 days median), 40 pigs were given a single 2 mL vaccination intramuscularly in the neck, using a 20 gauge, ¾ in. needle. Another 40 pigs were injected with 2 mL of normal saline as a control. Five pigs were allocated to a sentinel group, and these pigs were administered normal saline in the same manner as the control pigs.

The pigs were weaned one day after their vaccination. Upon weaning, the pigs were transported to isolation facilities for the pre-challenge and challenge phases of the study. Pigs from each group were comingled with approximately equal numbers of vaccinates and controls in each pen.

Prior to the challenge, the treatment groups were divided randomly into a subset of 25 pigs to be necropsied at 21 days post-challenge (dpc) (necropsy groups), and a subset of 15 pigs to be fecal sampled to determine *L. intracellularis* shedding during the post-challenge period (sampling groups, see Fig. 1). Randomizations were performed using the Microsoft Excel function Rand() [9]. Challenge occurred five weeks post-vaccination, when the pigs were approximately 8 weeks of age. The timing of necropsy was chosen based on published results of experimental infections [7], [10] and coincided with the peak time of fecal shedding and ileal lesions found in preliminary experiments with this challenge model (data not shown).

Prior to the start of the study, as well as prior to the challenge, all pigs were bled and sera were prepared. Pigs were bled again at the end of the test, at 21 dpc for the necropsy groups and at 51 dpc for the shedding groups.

### 2.4 Husbandry

Water was provided *ad libitum* to all animals. Feed met or exceeded the minimum nutritional requirements for animals of this age and complied with standard procedures for the site. During the pre-weaning phase, the piglets had access to the dam’s feed and water dispensers. Sow feed during this phase was a standard, non-medicated lactation diet. Feed was medicated with Mecadox (carbadox) at 50 g/ton, from the day after the vaccination when the pigs were weaned, until two weeks later (14 dpv). Carbadox is a quinoxaline antibiotic indicated for the control of swine dysentery and control of bacterial swine enteritis caused by *Salmonella choleraesuis*, but also effective against *L. intracellularis*, with a reported intracellular minimum inhibitory concentration of <0.5 µg/mL [11], [12]. Three weeks after weaning, mild loose stools were observed in some of the pens. All pigs were therefore treated for three days with gentamicin between 22 and 24 dpv. This medication was administered through the water supply, at 1 mg/pig/day. Two pigs were removed from the study at 9 and 12 dpv due to head-tilt and ataxia. Both pigs were allocated in the vaccinated necropsy group. Diagnostic work-ups resulted in diagnoses of bacterial meningitis and possible otitis media, considered unrelated to the vaccination.

### 2.5 Challenge

#### 2.5.1 Challenge material

At the time of challenge, the five sentinel pigs were necropsied and evaluated for evidence of *L. intracellularis* exposure as detailed in Section 2.8 Necropsy. The remaining 80 pigs were individually orally challenged by syringe into the back of the mouth with 25 mL of *L. intracellularis*-infected gut homogenate, containing approximately 9.3 Log_10_ of *L. intracellularis*, as determined by qPCR. The gut homogenate was prepared from intestines of pigs experimentally challenged with a virulent isolate obtained in 2008 from pigs in the United States displaying clinical signs of ileitis. Briefly, mucosa from guts of pigs with clinical signs of ileitis were collected by scraping, diluted in sucrose-phosphate-glutamate buffer, homogenized in a warren blender, trypsinized, aliquoted and frozen at −70 °C essentially as described previously [13].

#### 2.5.2 Scoring of clinical signs of ileitis

Following the challenge, all pigs were observed daily for clinical signs of ileitis, including body condition, alertness, appetite, and consistency of the feces. Fecal consistency was scored as normal, soft or watery (mild diarrhea), bloody or tarry (severe diarrhea).

### 2.6 Serology

Five to 10 mL blood was collected in serum separator tubes, and centrifuged as recommended by the manufacturer, usually for 20 min at 860*g*. Sera were collected and stored frozen at −20 °C or lower until testing by a commercial competitive ELISA as documented in Section 2.3 [14]. Results are reported as percent (%) inhibition. Per the manufacturer’s instructions, results below 20% are negative for *L. intracellularis* antibodies, between 20 and 30% are considered suspect, and 30% and higher indicate positive results.

### 2.7 Quantitative real-time PCR

Quantitative real-time PCR (qPCR) of ileal scrapings and fecal samples was conducted at the Iowa State University Veterinary Diagnostic Laboratory, according to previous standard methods [15]. Briefly, the region (nucleotide 583–680) of the 16S rDNA gene of *L. intracellularis* (GenBank accession # L157390) was monitored with the forward primer, 5′-GCGCGCGTAGGTGGTTATAT-3′, the reverse primer, 5′-GCCACCCTCTCCGATACTCA-3′ and the fluorescent probe 5′-FAM-CACCGCTTAACGGTGGAACAGCCTT-TAMRA-3′. The qPCR conditions were as described previously [15]. Results (Ct values) were reported as the cycle number where fluorescence exceeded the threshold value. All assays continued for 40 cycles. In order to calculate the amount of *L. intracellularis* present in the samples and to evaluate the treatment effect, Ct values were transformed to copies per reaction (c/rx) by assuming that 40 Ct represents absence of *L. intracellularis* DNA (0 c/rx), and assuming 100% efficiency of replication, resulting in a 10-fold increase of DNA for every 3.3 cycles reduction in Ct values. Calculations were therefore carried out as follows: c/rx = 2 ^ (40-Ct).

### 2.8 Necropsy

The sentinel pigs were necropsied at the time of the challenge, while the necropsy groups were sacrificed at 21 dpc. In each case, the ileum was opened longitudinally, and gross lesions of ileitis were scored. Mucosa were scraped using a glass slide into a 50 mL conical tube, and the collected material was snap-frozen on dry ice and stored at −70 °C until further processing such as the determination of the level of *L. intracellularis* in intestinal epithelial cells as measured by qPCR. Furthermore a tissue sample was fixed in buffered neutral formalin for microscopic evaluations as described below.

#### 2.8.1 Gross lesion scoring

Gross lesions were scored on the basis of the severity of mucosal thickening (0- normal, 1- slight edema/hyperemia, 2- moderate hyperemia/mucosal thickening, 3- severe mucosal thickening, 4- severe hemorrhaging/necrosis/fibrinous exudate). Scores 2–4 were considered indicative of clinical ileitis.

#### 2.8.2 Histopathology for *L. intracellularis*

Formalin-fixed tissues were submitted to the Iowa State University Veterinary Diagnostic Laboratory for standard histopathological examination following Haematoxylin-Eosin (H&E) staining to evaluate microscopic lesions, mainly proliferation, consistent with *L. intracellularis* (0- no lesions; 1- mild proliferation; 2- marked proliferative enterocolitis).

#### 2.8.3 PAS staining

For PAS staining, fixed tissues were deparaffinized and rehydrated, immersed in periodic acid and stained with Schiff’s reagent as described previously [6].

#### 2.8.4 Immunofluorescence

For immunofluorescence (IF) all the antibodies tested (Supplementary Table 1) except for anti-*L. intracellularis* antibody required heat-mediated antigen retrieval in citrate buffer at pH6, using a Histo5 Rapid Microwave Histoprocessor (Milestone) with the following protocol: high pressure treatment at 110 °C for 5 min (20 slides per sample). For *L. intracellularis* immunostaining, sections were incubated in proteinase K (DAKO UK Ltd., Ely, UK) for 10 min at room temperature, followed by two washes in phosphate buffered saline (PBS). Solutions of 0.10% Triton-X in PBS and 0.010% Triton-X in PBS were used to permeabilize the tissue for 10 min at room temperature.

All IF slides were incubated in 5% bovine serum albumin (BSA) in PBS at room temperature for 30 min. Excess blocking solution was removed and slides were incubated overnight at 4 °C with primary antibodies in 1% BSA/PBS at the dilutions stated in Supplementary Table 1. Negative controls were subjected to the same procedures but with no primary antibodies. Isotype antibody controls for the primary antibody for 5 sentinels and representative subsets of 3 controls and 3 vaccinates were also conducted (Supplementary data 1). Slides were washed twice in PBS before a one hour incubation at room temperature with secondary antibodies Alexa-Fluor-647 goat anti-rabbit (1:1000, Thermo Fisher Scientific) or Alexa-488 isothiocyanate (FITC)-conjugated goat anti-mouse Immunoglobulin G (IgG) (Fc specific) F(ab)2 fragment (1:1000, Sigma-Aldrich) diluted in 1% BSA/PBS. Slides were washed twice in PBS then counterstained with 6-diamidino-2-phenylindone (DAPI, 1:1000 in water, Thermo Fisher Scientific) at room temperature for 30 min. Slides were again washed twice in PBS and were finally mounted with Lab Vision PermaFluor aqueous mounting medium (Thermo Fisher Scientific).

Intestinal alkaline phosphatase (IAP) was monitored with Vector® Red Alkaline Phosphatase Substrate (Vector Laboratories, Cat. No. SK-5100) according to manufacturer instructions. Slides were stained with DAPI and mounted as described above. The presence of intestinal alkaline phosphatase was viewed with a Texas Red® filter.

All sections were observed using LSM700 confocal laser scanning microscope (Carl Zeiss) and 2–4 images per sections were captured and processed blind by a single operator using Zen Blue software (Carl Zeiss).

#### 2.8.5 Quantitative image analysis

For quantitative analysis of PAS staining for Fig. 6, images were acquired at 20× magnification from at least three random fields per condition (Sentinels, Controls, Vaccinates) and processed using Image J 1.49S as described previously [6], [16]. Briefly, the green channel was selected and the intensity threshold was adjusted to highlight goblet cells. Crypts were manually outlined and PAS signal was measured using the purple channel for four to six crypts per section and signal intensity was normalized to the section area.

For quantitative assessment of IF signals of Fig. 6, confocal images at 40× magnification were stained with appropriate antibody and recommended secondary fluorescent antibody (Supplementary Table 1). For *L. intracellularis* and Mucin 2 (MUC2), two to four random fields from each staining were analyzed using Image J 1.49S. Briefly, the spectrally opposite color channel was selected and intensity threshold was adjusted to highlight the marker. Next, the appropriate color channel (green or purple) was selected, and signal intensity was normalized to the field area and recorded. For cleaved caspase-3 (CASP3) and Calprotectin (CP), and also for IAP, signal intensity was normalized to the section area and then recorded.

### 2.9 Statistical analysis

Statistical analysis for the bacterial shedding data using qPCR, serological results and quantitative image analysis were carried out using 1-way ANOVA used after assessing normality of the data using SAS package with α = 0.05 [17]. Incidence data comparing the two treatments were analyzed by Fisher’s exact test (GraphPad Quickcalcs, http://www.graphpad.com/quickcalcs/). qPCR results were evaluated by the Mann-Whitney test (Minitab v 17). Scatterplots were generated using GraphPad Prism v 7.02.

## 3 Results

### 3.1 Reduction of signs of clinical disease in vaccinated pigs

All pigs were individually observed every other day for three weeks following vaccination for any evidence of adverse systemic or injection site reactions. No systemic adverse reactions to the vaccination were observed. Local injection site reactions were observed in 10 of 40 (25%) vaccinated animals, ranging in size between 1 × 1 cm and 4 × 4 cm (length × width). All local reactions resolved by 19 days post-vaccination (dpv) without intervention.

Signs of clinical disease were monitored during the challenge phase of the experiment. Severe diarrhea was observed in 16 of 40 animals in the control group (40%), but not in any of the 40 vaccinated animals. In the vaccinated group, 11 of 40 animals (28%) presented with mild diarrhea at any time during the post-challenge period. Loss of appetite, lethargy or ill thrift was recorded for four control pigs.

**Fig. 2:**
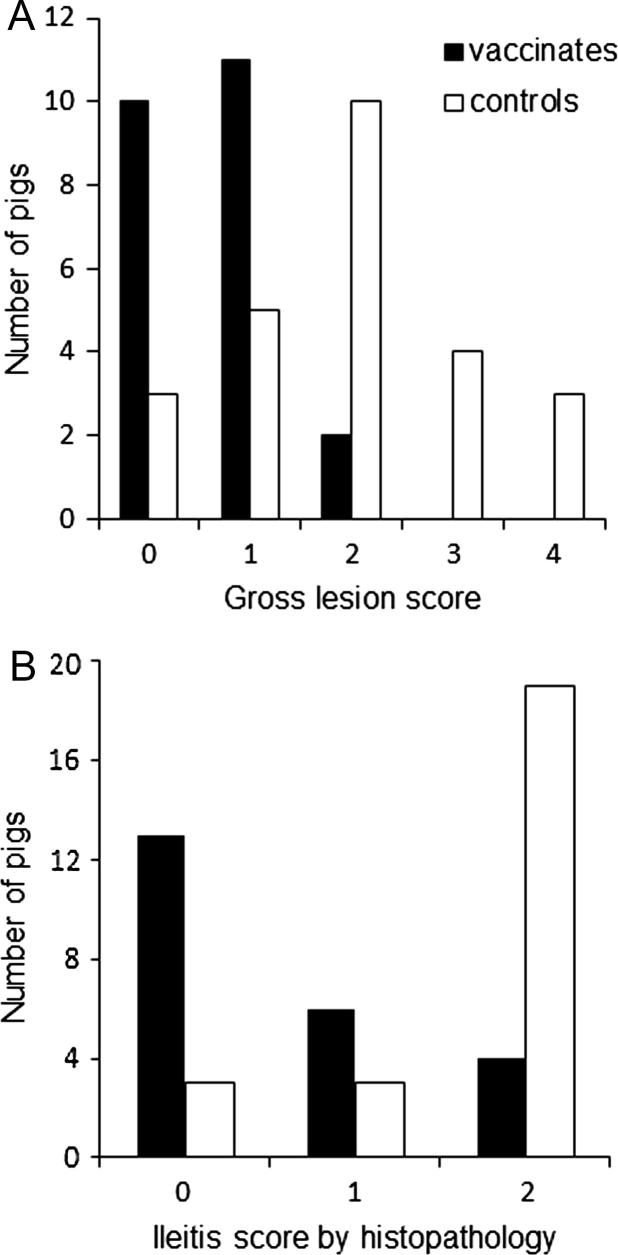
Gross lesion and histopathology scoring. (A) A subset of pigs was necropsied at 21 dpc. The ileum was removed and the mucosa was exposed, then scored on an ordinal scale (0–4) for increasing severity of gross lesions of ileitis, mainly mucosal thickening, hyperemia and fibrinous exudate. Scores 2, 3 and 4 were indicative for ileitis. The incidence of gross lesions of ileitis in the vaccinated group was significantly reduced compared to the control group (*p < .0001*). (B) Histopathology was performed on fixed ileum tissues from the pigs necropsied at 21 dpc. Tissues were scored on an ordinal scale (0–2) for increasing severity of histological lesions of ileitis. Scores larger than 0 were indicative for ileitis. The incidence of microscopic lesions of ileitis in the vaccinated group was significantly reduced compared to the control group *(p = .0018*).

Ileum gross lesion scoring is summarized in Fig. 2A. In the control group, 17 of 25 pigs (68%) were scored as positive (score >1), whereas only 2 of the 23 vaccinated pigs (9%) were scored as positive. Seven of the 25 control animals (28%) were given a score of 3 or 4, indicating severe ileitis, while none of the vaccinated pigs had scores higher than 2. The incidence of gross lesions of ileitis in the vaccinated group was significantly reduced compared to the control group (*p < .0001*).

The occurrence of clinical ileitis was also evaluated by histopathological examination, on an ordinal scale 0–2 (Fig. 2B). Of the 25 controls, 22 (88%) were positive (score >0), with 3 pigs presenting with score 1 and 19 pigs with score 2. Of the vaccinated group, 10 of 23 (43%) were positive for microscopic lesions. Overall the incidence of microscopic lesions of ileitis in the vaccinated group was significantly reduced compared to the control group *(p = .0018*).

### 3.2 Reduction of bacterial load in ileum of vaccinated pigs

**Fig. 3:**
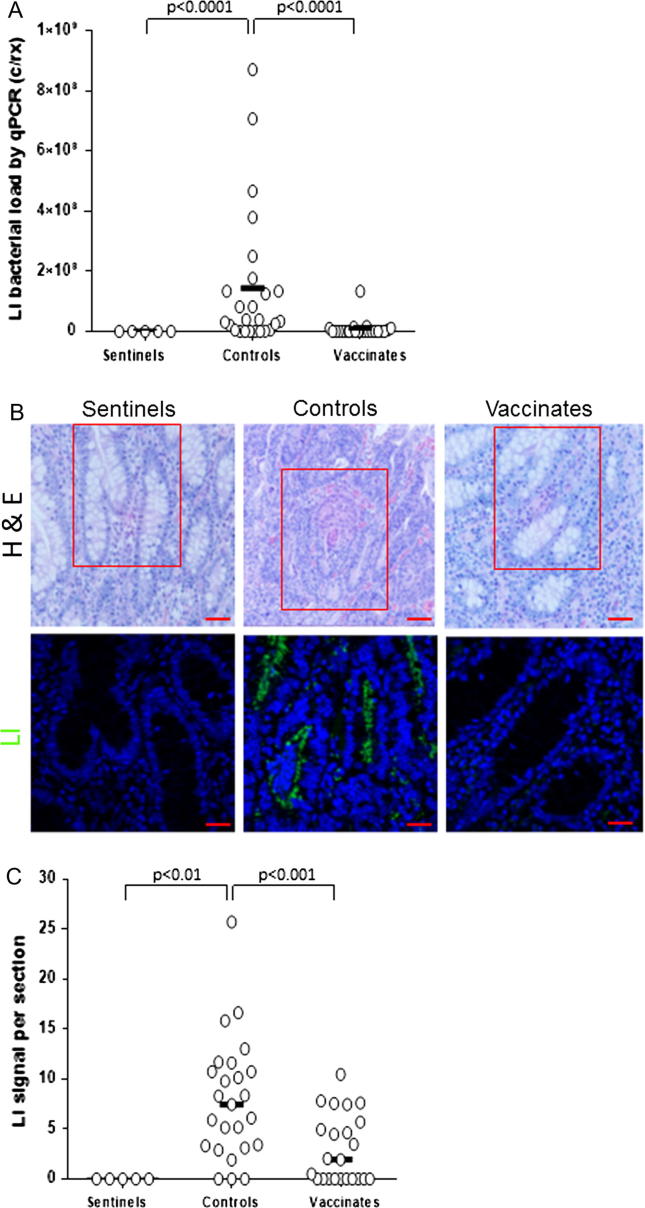
Quantification of *L. intracellularis* infection using qPCR and immunofluorescence. (A) qPCR was conducted to determine the amount of *L. intracellularis* DNA in mucosal scrapings collected at 21 days post challenge. qPCR results were reported as threshold cycle numbers (Ct) and were converted to copies per reaction (c/rx) as described in the text. Assays were concluded after 40 cycles. Group mean values are shown, and p-values when *p < .05*. (B) H&E and IF detection of *L. intracellularis* bacteria. H&E and IF assays were conducted in a blinded fashion on parallel sections. IF results using monoclonal VPM53 antibody, showing *L. intracellularis* bacteria (LI) in representative sentinel, control and vaccinate ileum samples 21 dpc. The presence of anti *L. intracellularis* antibodies (VPM53) was detected using FITC-conjugated secondary antibodies (green). Nuclei were counterstained with DAPI (Blue). Red box in H&E represents the same location on parallel section used to detect *L. intracellularis* bacterium. Scale bar (red line): 50 µm for H&E and 20 µm for LI. Signal intensity was determined as described in materials and methods section. (C) Scatterplot of *L. intracellularis* staining signal intensity obtained using Image J software for sentinel, control and vaccinate pig ileum crypts at 21 dpc. For statistical analysis between groups a 1-way ANOVA was used. Group medians are shown, and *p-values* where statistically significant differences were found (*p < .05*). Y-axis represents fluorescence signal intensity of *L. intracellularis* antigen staining per section. (For interpretation of the references to color in this figure legend, the reader is referred to the web version of this article, Supplementary Table 1.)

Colonization of the small intestines was evaluated in the necropsy group at 21 dpc by qPCR of the ileal scrapings and *L. intracellularis-*specific IF. Results are shown in Fig. 3. All animals were positive by qPCR on ileal scrapings (Fig. 3A); however, the average *L. intracellularis* DNA load of vaccinated pigs was significantly lower than that of the control pigs (geometric mean titer GMT: 5.0 × 10^4^ vs 1.1 × 10^7^ c/rx, *p < .0001*). Using IF of *L. intracellularis* specific antigen VPM53, intestinal crypt cells of controls harbored significantly greater bacterial load associated with enhanced cell proliferation than vaccinated pigs (*p < .001*) (Fig. 3B and C).

### 3.3 Vaccination induces anti-*L. Intracellularis* serum antibodies

**Fig. 4:**
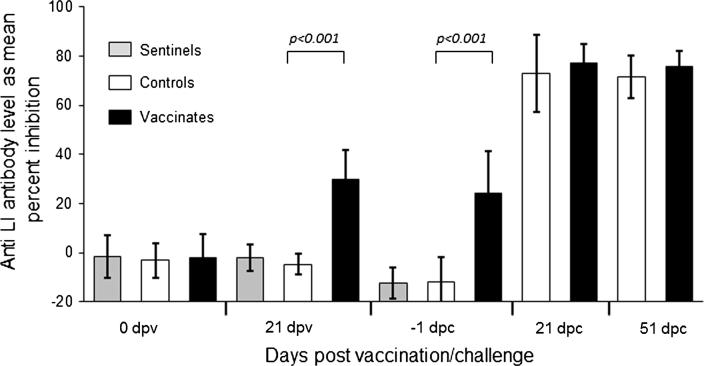
Antibody titers against *L. intracellularis* following vaccination and challenge. Group mean anti-*L. intracellularis* antibody levels were measured using an inhibition ELISA. Values lower than 30% inhibition are considered suspect or negative. The sentinels were removed at the time of the challenge, which took place at 5 weeks after the vaccinations. Group mean values and standard deviations are shown. Dpv: days post-vaccination, dpc: days post-challenge.

Vaccinated and control pigs were monitored for anti-*L. intracellularis* serological profiles using an inhibition assay (Fig. 4). Vaccinated pigs harbored significantly higher levels of anti-*L. intracellularis* antibodies prior to challenge than controls (*p < .0001*). Following challenge, all control pigs developed anti-*L. intracellularis* antibodies similar to vaccinated pigs as monitored at 21 and 51 dpc (*p > .05*).

### 3.4 Gut integrity is maintained in vaccinates

**Fig. 5:**
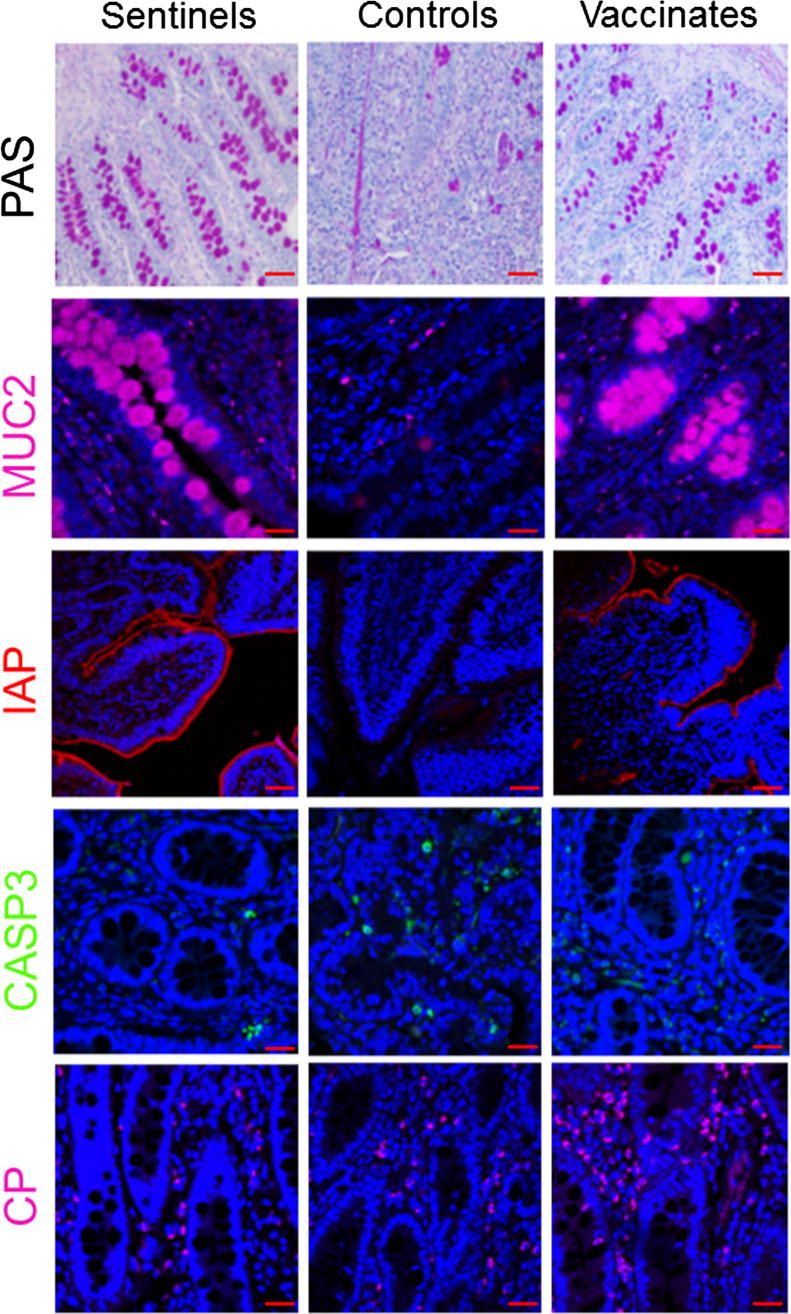
Assessing intestinal integrity of ileum 21 dpc in sentinel, control and vaccinate pig groups. Goblet cells were identified using PAS staining. IF was used to detect mucin-2 (MUC2), intestinal alkaline phosphatase (IAP), cleaved-caspase-3 (CASP3) and calprotectin (CP). Anti-MUC2 and anti-calprotectin antibodies were detected using Alexa-647-conjugated secondary antibody (purple). Anti-cleaved Caspase-3 antibody was detected using Alexa-488-conjugated secondary antibody (green). Intestinal alkaline phosphatase was detected as described in materials and methods. DAPI (blue) was used to counterstain the nuclei. Scale bar: 20 µM. Representative images are shown from animals with median results for each staining method and group. (For interpretation of the references to color in this figure legend, the reader is referred to the web version of this article.)

**Fig. 6:**
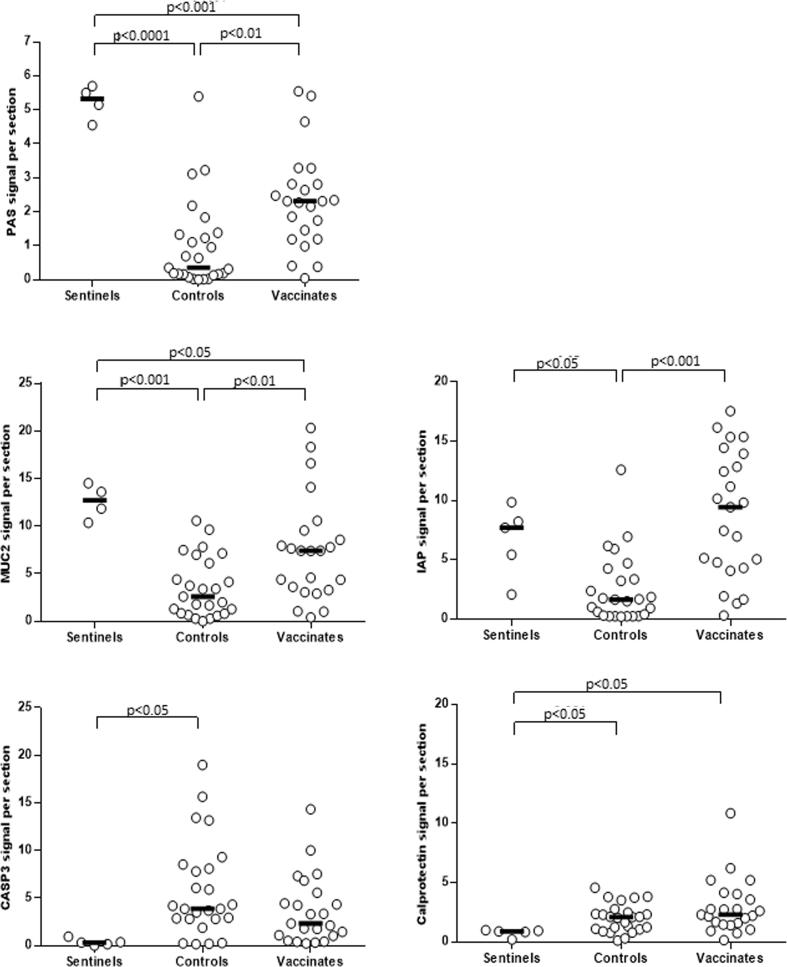
Scatterplot of PAS, IAP and IF signals for MUC2, CASP3 and CP. Scatterplot of staining signal intensities obtained using Image J software for sentinel, control and vaccinate ileum crypts at 21 dpc. Image quantification was performed as described in materials and methods section. For statistical analysis between groups a 1-way ANOVA was used as previously described. Group median values and *P-values* are shown when *p* < *.05*. For MUC2 and PAS staining 4 sentinels instead of 5 were used because one paraffin-block was missing at the time of the staining. Missing pig section was not assessed because of the relatively small spread between data. Y-axis represents fluorescence signal intensity of the marker staining per section.

To assess the integrity of the ileum tissues collected at 21 dpc, a range of intestinal cell lineage markers was evaluated and quantified. In particular, molecular markers that identify secretion (PAS signal and Mucin2, MUC2), absorption and homeostasis (Intestinal alkaline phosphatase, IAP), apoptosis (Cleaved-caspase3, CASP3) and immune cell distribution (Calprotectin, CP) were monitored (Figs. 5 and 6) [6], [16], [18], [19], [20]. In the vaccinated group (n = 23) the level of PAS, MUC2 as well as IAP was significantly higher than controls (n = 25; *p < .01, p < .01, p < .001, respectively)*. IAP expression in vaccinated pigs was similar to the level found in the sentinels (n = 5; *p > .05*)*.* Expression of apoptotic marker CASP3 was significantly up-regulated in controls (*p < .05*) pigs compared to the sentinels. CP, a marker associated with neutrophil infiltration, was expressed at a higher level compared to the sentinels, in both controls (*p > .05*) and vaccinates (*p < .05*).

### 3.5 Fifteen-fold reduction of *L. intracellularis* fecal shedding in vaccinates

**Fig. 7:**
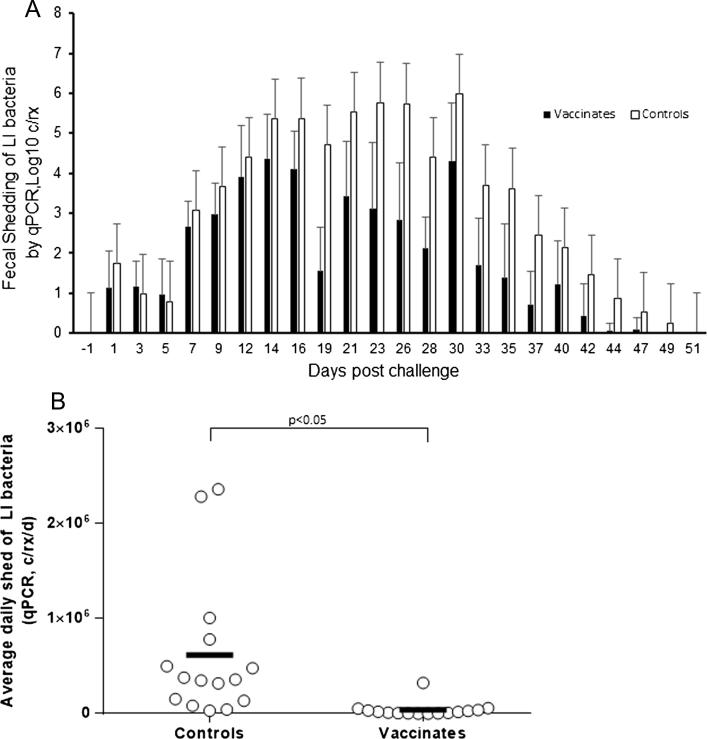
*L. intracellularis* fecal shedding. (A) Daily amount and standard deviation of *L. intracellularis* (LI) fecal shedding as determined by qPCR on fecal samples collected three times weekly until resolution of all shedding. (B) The daily average amount of *L. intracellularis* shed during the 51 day post-challenge observation period. Group mean values are indicated, and p-values when *p < .05*.

Fecal shedding was monitored in a subset of the vaccinated (n = 15) and control (n = 15) animals using qPCR over 51 dpc (Fig. 7A). The mean daily average amount of *L. intracellularis* shed during the 51 dpc was 6.2 × 10^5^ copies/reaction/day for the control group, and 4.1 × 10^4^ per day for the test vaccine animals, representing a 15-fold reduction (*p < .05*, Fig. 7B). Overall, the median duration of shedding was significantly reduced in the vaccinated group by 4.4 days (*p < .05*, data not shown). The median time point where maximum shedding occurred was also earlier in vaccinates (14 dpc versus 23 dpc), although this difference was not statistically significant (*p > .05*).

## 4 Discussion

In this study, we have assessed the efficacy of a novel inactivated, injectable whole-cell bacteria vaccine against *L. intracellularis* – Porcilis Ileitis. We found that this adjuvanted *L. intracellularis* vaccine was safe in pigs when administered under typical field conditions at three weeks of age prior to weaning. Following challenge, signs of severe clinical ileitis were observed in 40% of the controls, but not in the vaccinated pigs. Protection of vaccinates was further confirmed by gross observation of the ileum, as well as histopathology. For each of these parameters, significantly more control animals were affected than vaccinated animals. Taken together these results indicate that the vaccine reduced both the incidence and severity of clinical signs.

At the time of vaccination nearly all of the animals tested negative for anti-*L. intracellularis* antibodies by ELISA, although three of 85 pigs had test results which were classified as ‘suspect’ by the manufacturer’s criteria, most likely as a result of low levels of maternally-derived antibodies against *L. intracellularis*. The ‘suspect’ animals were included in the analyses. Vaccinated pigs displayed an antibody response in the majority of pigs, in spite of the vaccine regimen consisting of only a single dose. At three weeks post-vaccination, 75% of vaccinates were classified as suspect or positive. No apparent correlations were found between sero-response following vaccination and outcome parameters of the challenge, such as clinical signs, gross lesions, microscopic scores or PCR results (data not shown). This suggests that at least part of the protective mechanism of this inactivated, parenteral vaccine may be through induction of cell-mediated immunity (CMI) as previously described for other bacterial pathogens, such as *Mycoplasma hyopneumoniae* [21], [22], [23]. Further investigation is required to assess if this is the case.

Our study clearly showed that reduced clinical signs in vaccinates coincided with a reduced bacterial load and increased ileum integrity at 21 dpc, compared to nonvaccinated controls. The bacterial load was significantly (200-fold) reduced in the terminal ileum as determined by qPCR, and was significantly lower when examined by *L. intracellularis-*specific IF. Bacterial loads were also significantly lower in the feces of vaccinated animals, both in terms of reduced severity (bacteria shed per day) and in reduction of the duration of fecal shedding. Reduced proliferation of enterocytes in vaccinated pigs compared to controls, as demonstrated by H&E staining of ileum tissues, coincided with significantly greater numbers of MUC2-expressing goblet cells and IAP-expressing brush border cells than controls indicating a restoration of these essential intestinal cell lineages [6], [16], [18], [19], [20]. Similarly, the level of cleaved-CASP3 in lumen of the ileum of vaccinated pigs was, at least in part, reduced to the level observed in sentinel pigs. This may indicate a possible reduction in apoptosis mechanisms as previously described [16]. In contrast, we found that the induction of CP, a marker of neutrophil and macrophage infiltration, was still noticeably expressed in the ileum of vaccinated pigs, perhaps indicating that innate immune cells were actively patrolling the lamina propria at 21 dpc [24]. Taken together, the monitoring of these intestinal cell lineages and immune markers supports an immunization mechanism whereby the vaccination not only prevented infection by *L. intracellularis* of the crypt cells, but also helped preserve near normal mucosal architecture and homeostasis.

According to Johansen et al. [25], diarrhea is a significant risk factor for low growth rate in pigs, and a 10-fold increase in fecal load of *L. intracellularis* increases the odds ratio for low growth rate by 2. In this study, fecal shed in the vaccinates was reduced 15-fold compared to the controls. Although this study did not record growth parameters, the vaccination can be expected to have prevented the negative impacts of *L. intracellularis* infection on the economic parameters of this cohort of pigs.

The use of a killed Lawsonia vaccine in this study allowed the administration of antibiotics to the pigs around the time of the vaccination. The vaccine was compatible with the use of carbadox administered in the feed as early as one day after vaccination, as demonstrated by the protection that was shown against virulent challenge compared with non-vaccinated control pigs.

In conclusion, the data demonstrated that this inactivated, adjuvanted *L. intracellularis* vaccine was safe in pigs when administered under typical field conditions as a single, 2 mL dose at three weeks of age prior to weaning. The study demonstrated that the vaccine reduced both the incidence and severity of clinical signs of ileitis due to *L. intracellularis*, reduced colonization by *L. intracellularis*, and reduced the severity and duration of fecal shedding of *L. intracellularis*. The study also found that gut function in the face of *L. intracellularis* infection was improved following vaccination.

## Conflict of interest

This study was funded by Merck Animal Health, Madison, NJ USA. The animal trial was designed by FR and ES. End-of-study lesion scoring was conducted by FR while blinded to the grouping. M-HP, AA and TA-A declare no conflict of interest.
