# Supplementary material for: A novel inactivated vaccine against Lawsonia intracellularis induces rapid induction of humoral immunity, reduction of bacterial shedding and provides robust gut barrier function
Source: Vaccine. 2018 Mar 7;36(11):1500–8. doi: 10.1016/j.vaccine.2017.12.049 (PMC5846845; doi:10.1016/j.vaccine.2017.12.049)
Supplement: Supplementary data 1 [file mmc1.docx]

Supplemental data

Supplementary Table 1

| Antibodies | Source of antibody (Host) | Provider | Dilution | Remarks |
| --- | --- | --- | --- | --- |
| *L. intracellularis* (LI) | Mouse | University of Edinburgh | 1:400 | Targeting cell surface transporter of *L. intracellularis* |
| Mucin 2 (MUC2) | Rabbit | Abcam | 1:1000 | Secretory Mucin (goblet cell marker) |
| Calprotectin (CP) | Rabbit | SantaCruz | 1:1000 | Monocytes and neutrophils |
| Cleaved caspase-3 (CASP3) | Mouse | Cell signalling | 1:400 | Apoptotic cells |
| Primary rabbit IgG isotype control | Rabbit | Thermofisher | 1:1000 | Isotype control for CP and MUC2 immunofluorescence signal |
| Primary mouse IgG isotype control | Mouse | Thermofisher | 1:1000 | Isotype control for CP and MUC2 immunofluorescence signal |
| Alexa-Fluor-647 goat anti-rabbit | goat | Thermofisher | 1:1000 | Anti-rabbit secondary antibody |
| Alexa-488 isothiocyanate (FITC)-goat anti-mouse Immunoglobulin G (IgG) (Fc specific) F(ab)2 fragment | goat | Sigma-Aldrich | 1:1000 | Anti-mouse secondary antibody |
